# The Global Burden of Disease Assessments—WHO Is Responsible?

**DOI:** 10.1371/journal.pntd.0000161

**Published:** 2007-12-26

**Authors:** Claudia Stein, Tanja Kuchenmüller, Saskia Hendrickx, Annette Prüss-Űstün, Lara Wolfson, Dirk Engels, Jørgen Schlundt

**Affiliations:** 1 World Health Organization, Geneva, Switzerland; Swiss Tropical Institute, Switzerland

## Abstract

The Global Burden of Disease (GBD) concept has been used by the World Health Organization (WHO) for its reporting on health information for nearly 10 years. The GBD approach results in a single summary measure of morbidity, disability, and mortality, the so-called disability-adjusted life year (DALY). To ensure transparency and objectivity in the derivation of health information, WHO has been urged to use reference groups of external experts to estimate burden of disease.

Under the leadership and coordination of WHO, expert groups have been appraising and abstracting burden of disease information. Examples include the Child Health Epidemiology Reference Group (CHERG), the Malaria Monitoring and Evaluation Reference Group (MERG), and the recently established Foodborne Disease Burden Epidemiology Reference Group (FERG). The structure and functioning of and lessons learnt by these groups are described in this paper.

External WHO expert groups have provided independent scientific health information while operating under considerable differences in structure and functioning. Although it is not appropriate to devise a single “best practice” model, the common thread described by all groups is the necessity of WHO's leadership and coordination to ensure the provision and dissemination of health information that is to be globally accepted and valued.

## Introduction

Borrowing the words of the New Testament Apostle Paul, Samuel H. Preston stated that “before 1990, the global disease landscape…was perceived through a glass darkly” [1]. Indeed, the *Global Burden of Disease (GBD) 1990* series [2] was a landmark publication that constructed an internally consistent global overview of morbidity, disability, and mortality burden for some 130 diseases and conditions. Frustrated by fragmented, incomplete, incomparable, and often advocacy-driven health information, the authors of the GBD 1990 synthesized a plethora of data and health measures into a single health metric, the so-called disability-adjusted life year (DALY), thus permitting policy makers to directly compare the burden of different diseases, set priorities, and evaluate the cost-effectiveness of their interventions.

The World Health Organization (WHO) was a major partner in the GBD 1990 study and officially adopted its approach for reporting on health information in the late 1990s. Soon individual technical units and programs within WHO used and further developed the method and built collaborations with external experts to publish disease burden estimates beyond the “classic” GBD cause list [3]–[5]. Since then, the tapestry of burden of disease assessments has continued to grow, and major collaborative initiatives have emerged to this effect at WHO in recent years.

In this paper, we describe WHO's responsibility in global burden of disease assessment and summarize major ongoing and planned activities in the synthesis and appraisal of existing and new global burden of disease data put forward by WHO. We explore the critical role of WHO in these efforts and outline how areas of collaboration, partnership, and synergy can be forged to provide credible and meaningful global health statistics. However, the function and activities of the WHO department specifically dedicated to health information, including the hosting of the global partnership of the Health Metrics Network [6], are sufficiently extensive to be dealt with in a separate publication in this series [7].

## Background

The GBD approach was developed in the 1980s with the commissioning of cost-effectiveness analyses by the World Bank. The results of this effort were first published in the *World Development Report 1993* [8] and the *Disease Control Priorities in Developing Countries* project [9]. Since adopting the GBD approach in its health reporting, WHO has not only undertaken a major review of the GBD 1990 with its GBD 2000 publications [10], but also provided annual updates in the annex tables of the *World Health Report* [11]. Moreover, in collaboration with external scientists, WHO developed creative new methodologies for the assessment of disease burden resulting from risk factors [12]. The latter included a widely publicized contribution estimating the GBD from environmental factors such as unsafe water and sanitation, climate change, unsafe sex, and lead exposure, among others. The DALY approach brought new knowledge to the public health community, which was particularly evident in the *World Health Report 2001—Mental Health: New Understanding, New Hope* [13]. This publication quantified for the first time the “silent burden” of mental disorders by identifying depressive disorders as the leading cause of disability among men and women world-wide. A succinct summary of the GBD study and its evolution is given by Mathers et al. [7].

The technical approach of the GBD is complex, both in concept and in application, and the interpretation of results requires detailed methodological knowledge. As WHO increasingly used these approaches, it was urged by Member States and international scientists to provide more transparency in the underlying methods and inputs used [14]. This was a particular concern in the wake of the *World Health Report 2000,* which controversially published a league-table of countries' health systems performance using complex mathematical models [15]. In 2005, WHO therefore convened an international high-level advisory panel on health statistics [16], which recommended that WHO work with external reference groups to ensure accuracy and transparency of estimates. The panel made a number of detailed recommendations in the areas of data collection and reporting, comparability of statistics between countries, and the provision of time series of epidemiological information, as well as the reporting of uncertainty ranges, especially when providing country-level estimates. Moreover, the panel advised WHO to make major efforts to support the in-country application of estimation procedures, including the simplification of tools and methods and building of national capacity. The high-level panel echoed statements previously made by Burden of Disease champions, including Christopher Murray, who expressed hope that WHO would advance the GBD methods [17]. The panel particularly emphasized WHO's constitutional and legitimate link with its Member States [16], which mandates the reporting of health data, capitalizes on the convening power of WHO to reach consensus and its leadership to develop and harmonize methods and tools for health information, in collaboration with relevant partners.

Informally, the process of drawing on external experts to derive burden of disease estimates had already been spearheaded through individual WHO units, first and foremost the department of Child and Adolescent Health, which established the Child Health Epidemiology Reference Group (CHERG) in 2001. The CHERG, which is composed of external advisers in the field of child health epidemiology, estimated child mortality burden for several major causes and published two acclaimed series in *The Lancet* [18]. This model was followed by other programs, including the Malaria Monitoring and Evaluation Reference Group (MERG) and the Initiative for Vaccine Research, which hosts a Steering Group to review and develop estimates of vaccine-preventable diseases. WHO's department of Neglected Tropical Diseases (NTDs) recently established a Strategic and Technical Advisory Group (STAG) charged with leading burden of disease efforts, among other tasks, while the department of Food Safety, Zoonoses and Foodborne Diseases has instituted a Foodborne Disease Burden Epidemiology Reference Group (FERG) to estimate the global burden of foodborne diseases from microbiological and chemical causes.

As these different WHO advisory reference groups evolve, so does the complexity of their structures and tasks, partnership configurations, expert enrollment practices, and funding mechanisms. The following paragraphs outline the different developments and models. This review, however, does not claim to be an exhaustive analysis. Recognizing that we cannot review all projects in this context, our paper concentrates on the major initiatives of interest to readers with a focus on NTDs.

## From CHERG to FERG and Beyond

### Child health

In June 2001, the CHERG was formally established in response to increasing demands for better health information and methods for estimating cause-specific mortality among children less than five years of age. Its aim was to provide data that could support priority setting and decision making for the implementation of child health interventions. Until then, researchers and institutions were using a variety of different estimation and death classification methods. Moreover, estimates developed up to that point were not generally based on data obtained from systematic reviews, and transparent procedures were not always being used. Recognizing that partnerships with external entities were critical to arrive at unbiased epidemiological estimates, the CHERG was created as a group of experts external to the United Nations (UN) system and guided by a small group of “core” members. It operates through ad hoc working groups that address specific issues, while at the same time it depends on active participation, consultation, and inputs from WHO technical staff as well as from other UN agencies such as the Joint United Nations Programme on HIV/AIDS (UNAIDS) and the United Nations Children's Fund (UNICEF).

When the CHERG was founded, its mandate was to review and improve data collection, methods, and assumptions underlying estimates of the major causes of under-five morbidity and mortality, including pneumonia, diarrheal diseases, malaria, and measles, as well as the main causes of death in the neonatal period. The role of undernutrition as an associated cause of deaths was also estimated. The CHERG commenced its work with clear goals and expected outcomes in mind but organized its terms of reference as it evolved. This meant that it could easily adapt to changing needs, but also contributed to some confusion about the roles and responsibilities of individual group members. The group has been highly productive and published its finding in two acclaimed series in *The Lancet* as well as other high-impact journals [18], thus maintaining the policy debate on child mortality at the highest levels.

### Malaria

The MERG has a wider scope than CHERG, acting as advisory body to the Roll Back Malaria (RBM) partnership on all matters pertaining to monitoring. It was formed in response to a RBM five-year evaluation in 2003, which called for the establishment of a reference group for periodic consultation on technical issues. The main focus of the MERG has been on developing of consensus on national and global indicators, data collection, and analytic approaches related to disease burden as well as coverage of interventions in order to document progress in scaling up malaria prevention and control efforts. The MERG is comprised of 10–15 core members and external experts who are invited on a temporary basis. Six task forces within MERG are dealing with and publishing on specific monitoring and evaluation topics, including individual task forces on mortality trends, on indicators and estimation of malaria morbidity, and on survey and indicator guidance, as well as a task force on assessing the economic impact of malaria. The MERG continues its work and has recorded significant results, including the development of effective consensus building mechanisms for core monitoring and evaluation activities and data collection methods.

### Environmental risks

Largely as a result of the collaborative efforts leading to the *World Health Report 2002—Reducing Risks, Promoting Healthy Life* [19], which examined the disease burden resulting from a variety of risk factors, the WHO Department of Public Health and Environment continues its work with a network of external collaborators on the burden of environmental risks through either updating the 2002 information or examining new environmental and occupational risk factors [20]. The latest update in this series [20] has recently won an award for excellence in the British Medical Association's annual Medical Book Competition [21]. Rather than establishing a defined working group, the unit opted for a theme-based approach where individual experts are enrolled for specific tasks on the basis of their international expertise to deliver burden of disease assessments. All work submitted is subsequently peer reviewed and screened by WHO to ensure that it conforms to the agreed methodology. Examples include the estimation of disease burden from contaminated sharps injuries in health care workers [22] (about 80,000 new hepatitis B or hepatitis C infections and 1,000 new HIV infections each year), and excessive ultraviolet radiation exposure [23] (leading to about 60,000 deaths per year).

Since 2003, the department has also been developing guides to assist countries in the estimation of their national (or local) burden of disease estimates. This “Environmental Burden of Disease Series” counts 10 guides to date covering specific environmental or occupational risks that can be applied to the national or sub-national level (including outdoor air pollution, indoor air pollution from solid fuel use, occupational airborne particulates, and occupational noise, among others). Another six guides are currently in preparation. In addition, the group has just recently launched 192 country profiles on environmental burden of disease, which are first estimates of national burden from various environmental risks.

### Vaccine-preventable diseases

WHO currently estimates that around 2.5 million children die each year of diseases that are preventable by vaccination [24]. The increasing pressure to have robust, annual estimates of the burden of vaccine-preventable diseases—both by disease and by year, including estimates of disease currently prevented by vaccination—has led to the WHO Initiative for Vaccine Research creating a new advisory group known as QUIVER (Quantitative Immunization and Vaccination Related-Research). This group is mandated to oversee the continual development and improvement of methods and data to evaluate the burden of vaccine-preventable diseases. It is also charged with setting standards for economic evaluation and the development of tools for assisting evidence-based decision making at the country level. QUIVER consists of 12 members and is assisted by a WHO secretariat that meets every two months to ensure progress is being made.

The disease burden estimates are based on models [25]–[28] described in both peer-reviewed and other publications and relying on immunization coverage and surveillance data collected through WHO's Vaccine-Preventable Diseases Monitoring System [29]. As more and more vaccines become suitable for developing country use, and as more funding is available to support the purchase of such vaccines, particularly through the Global Alliance for Vaccination and Immunization (the GAVI Alliance) (http://www.gavialliance.org/), the need for robust, country-level estimates of disease burden for each disease is increasing.

### Neglected tropical diseases

One of the most recent initiatives aimed at delivering burden of disease estimates has been established in the area of NTDs. In April 2007, a high-level STAG on NTDs was convened by WHO following a widely distributed call for experts. Recognizing the urgent need to reassess the largely underestimated burden caused by the NTDs, the STAG is charged with providing objective scientific advice to WHO in the area of burden of disease assessment. The overall scope of this group, however, is considerably wider than that of any other forum described in this paper. In addition to burden of disease estimation, the STAG advises on prevention and control of NTDs, including impact assessments and cost-effectiveness analyses. It also seeks to enhance collaboration and intervention implementation between NTD experts and other relevant groups, including the Commission on Social Determinants of Health and the United Nations Development Programme (UNDP)/World Bank/WHO/UNICEF Special Programme on Tropical Diseases Research. The STAG had immediate and high visibility through early involvement of scientific press in the process [30], ministerial participation in its first meeting, and through the commitment of an advocacy “champion” in the person of His Royal Highness, the Prince Abdulaziz Ahmad Al Saud of Saudi Arabia, himself a sufferer from the complications of one of the NTDs, namely trachoma. The STAG is taking up its work through a number of working groups and seeks to publish a peer-reviewed article describing the WHO NTD strategy and research priorities in the free-access scientific literature.

One of the NTDs that has recently received concerted WHO attention is leptospirosis, a disease with a significant health impact in many parts of the world, particularly in tropical countries in the Americas and Asia. It is mostly regarded as an epidemic disease with large visible outbreaks associated with floods. However, the burden of endemic leptospirosis is thought to be very significant for people living in rural areas involved in farming and urban area settlements with poor sanitation, including slums [31]. Special attention will therefore be devoted to this disease through the Leptospirosis Burden Epidemiology Reference Group (LERG). The group has issued a call for advisers in the scientific press and on WHO's Web site [32], and will be composed of epidemiological experts in the areas of zoonotic diseases, burden of disease methodologies, disease modeling, and clinical medicine. The initiative will also assist countries in conducting a national burden study of leptospirosis, preferably in conjunction with studies on other relevant NTDs.

Funding mechanisms for the initiatives described in this paper vary enormously and range from WHO internal resourcing to government funding, as well as support entirely provided by foundations. The individual funding mechanisms, as well as a summary of the purpose, structure, and procedures of the major burden of disease activities, are summarized in Table 1.

**Table 1:** Purpose, Structure, and Procedures of Formalized WHO Burden of Disease Activities

Group	Purpose	Formal Terms of Reference	Structure and Functions	Expert Enrollment	Products	Time Frame	Funding	Impact
**CHERG** (http://www.who.int/child-adolescent-health/publications/pubCNH.htm)	To estimate cause-specific morbidity and mortality in children under 5 years	No formal ToR available. CHERG is to • provide external technical guidance to WHO for the development and improvement of child health epidemiological estimates • address general methodological issues	• Core group plus working groups addressing specific disease groups/themes • Active participation and inputs from WHO technical staff and other UN agencies	Experts selected informally through peer nomination for internationally recognized expertise	Related to pneumonia, malaria, diarrheal diseases, multi-cause model, under-nutrition, co-morbidity: • Publications in public health journals (incl. series in *The Lancet* on child survival and on newborn health) • Technical reports • Contributions to World Health Reports	2001–2005	The Bill and Melinda Gates Foundation	• Increased public awareness and high-level policy debate
**MERG** (http://www.rollbackmalaria.org/merg.html)	To develop effective monitoring and evaluation mechanisms for the RBM Partnership	Formal ToR available (http://www.rollbackmalaria.org/partnership/wg/wg_monitoring/docs/tor_MERG.pdf). MERG is to • develop and provide technical guidance on selection and definition of indicators for national, inter-country, and global reporting	• Core group plus six task forces • External consultants (as required) • Observers	• Members are invited by the RBM Secretariat based on clear selection criteria	• Africa Malaria Report 2003 • World Malaria Report 2005 • RMB MERG Guidance Notes and Guidelines • Global malaria database	Since 2001		
**WHO's Programme on Quantifying Environmental Health Impacts** (http://www.who.int/quantifying_ehimpacts/en)	To provide morbidity, mortality, and DALY estimates for selected diseases from environmental risks	Generic ToR available. Work is peer reviewed and edited by WHO to ensure compliance with agreed methodology	No formal group—individual expert enrollment for specific tasks	Experts selected informally through peer nomination for internationally recognized expertise	• Guides for countries to conduct burden assessments • Global assessments of disease burden from environmental risks • 192 country profiles for environmental burden of disease	Since 2000	• Seed funds through USEPA • Selected donors contributing to their risk factor of interest	Achieved impact: • Public awareness • Difficult to measure impact in the countries/on policies
**QUIVER**	To provide annual estimates of the burden of vaccine-preventable diseases	Formal ToR available (http://www.who.int/vaccine_research/TOR_QuantitativeSC_Dec1.pdf). QUIVER is to oversee the continual development of methods and data to monitor the burden of vaccine-preventable diseases	• Core group and Task Teams for specific projects • External consultants (as required) • Observers (representative of the partner organizations)	• Public call for experts to serve for an initial term of one year with renewal • Members appointed by Director of WHO department Initiative of Vaccine Research	• Reports on disease burden estimates for vaccine-preventable diseases • Standards for economic evaluation in immunization • Tools for assisting evidence-based decision making at country level	Since 2007	• GAVI, CDC, and the Govern-ment of Japan (partly linked to specific projects)	
**NTD STAG** (http://www.who.int/neglected_diseases/stag/en/index.html)	To effectively prevent and control NTDs and assess socioeconomic impact	Formal ToR available (http://www.who.int/neglected_diseases/stag_annex_ok.pdf). NTD STAG is to • advise WHO on overall global policies/strategies ranging from monitoring and implementation to delivery • facilitate and monitor the coverage of interventions for the control of NTDs • document its socioeconomic benefits	• Core group plus sub-working groups addressing specific issues • Active participation and inputs from WHO technical staff and other UN agencies	• Public call for experts to serve for an initial term of two years on NTD STAG • WHO-internal selection of experts • Nominations approved by the DG	• Global report on global burden and socioeconomic impact of NTDs • Peer-reviewed papers	Since 2007	• WHO, CDC • Other external donors/foundations have been solicited	Expected impact: • Engage non-health sectors (including economic) in NTD control • Affect policy changes
**LERG** (http://www.who.int/zoonoses/diseases/lerg/en/index.html)	To obtain global epidemiological estimates on leptospirosis	Formal ToR available. LERG is to collate, appraise, and disseminate burden of leptospirosis estimates	• Core group of experts	• Public call for advisers • WHO-internal selection of experts • Nominations approved by the DG	• Global report on leptospirosis morbidity and mortality • Publications in peer-reviewed journals	2007–2010	• WHO • Govern-mental donors • Foundations will be solicited	Expected impact: • Strengthening of prevention and control measures
**FERG** (http://www.who.int/foodborne_disease/burden/en/index.html)	To provide reliable burden of disease estimates to enable policy makers and other stakeholders to set appropriate priorities in the area of food safety	Formal ToR and Standard Operating Procedures available. FERG is to • review epidemiological data on foodborne disease burden • identify technical gaps and priorities for research activities • report on burden of foodborne disease estimates	• Core group plus Task Forces addressing specific disease and causes • External experts (as required) • Active participation and inputs from WHO technical staff and other UN agencies	• Public call for advisers to serve on FERG for one year with renewal • WHO-internal selection of experts • Nominations approved by the DG	• Global report on morbidity, disability, and mortality from foodborne diseases • Peer-reviewed journal series on foodborne disease burden • Country guidelines and protocols for burden of foodborne disease assessments	2007–2011	• WHO • CDC • Governments and foundations have been solicited	Expected impact: • Strengthening of prevention and control measures • Capacity building at country level for foodborne disease burden assessment

DG, Director-General; ToR, Terms of Reference; USEPA, US Environmental Protection Agency

As is evident from Table 1, the groups described above constitute a very varied tapestry of burden initiatives, often reflecting established practice in the way disease programs have worked (including their verticality or horizontality), use of procedures that permit ease and speed of obtaining results, and a natural evolution of initiatives that started small. Two of the burden activities aiming at similar results but standing in noticeable contrast to each other when examining their structure, functions, and operational level are CHERG and NTD STAG (Table 2). While the trail-blazing CHERG was established from the “bottom up”, avoiding formal terms of references and a call for advisers and thus benefiting greatly from flexibility and speed, it opened itself up to potential conflicts of interest among members and potential criticism of lack of transparency as members were not selected through an open process. The NTD STAG was established using the opposite “top down” approach, where high-level political commitment (including a well-known public advocate) was sought early on in the process and the scientific press were timely observers. This ensured much-needed support from WHO Member States and extensive media coverage at the launch, enhancing opportunities for fund-raising, dissemination of results, and implementation at country level. The potential risks inherent in this approach are the politicisation of science and loss of scientific focus.

**Table 2:** In-Depth Case Studies: CHERG and NTD STAG

Group	Strengths	Opportunities	Lessons Learnt	Potential Risks
CHERG	• Initially established below formal organizational radar screen • Small and non-bureaucratic • High-level technical products with publication in high- impact journals	• Members are well embedded in the scientific community • Good momentum through MDGs	• Members and chair must specifically avoid conflicts of interests	• Conflicts of interest may influence results
NTD STAG	• High political visibility and buy-in • Charged with multiple tasks, including burden of disease assessment	• Early involvement of scientific press • Advocacy champion		• Political influence on science • Moving slowly • Loss of focus

### Foodborne diseases

Foodborne diseases encompass a wide spectrum of illnesses and are an important cause of morbidity and mortality worldwide. Diarrheal diseases alone—a considerable proportion of which are foodborne—kill an estimated 1.8 million children every year world-wide. Although most of these diarrheal deaths occur in poor countries, foodborne diseases are not limited to developing countries nor to children. It is estimated that in the United States, foodborne diseases result in 76 million illnesses, 325,000 hospitalizations, and 5,000 deaths each year [33]. The full extent of the burden and cost of unsafe food, however, is currently still unknown. Data from developing countries where populations are particularly exposed to contaminated environments are especially scarce. Without concerted action to estimate and reduce the burden of foodborne diseases, international efforts to achieve the Millennium Development Goals (MDGs), including the overarching goal of poverty reduction, will be jeopardized, particularly those goals relating to children and the poor.

Although several international initiatives are under way, no precise and consistent global information exists to date. WHO's Department of Food Safety, Zoonoses and Foodborne Diseases (FOS) therefore launched an initiative to estimate the global burden of foodborne diseases from all major causes, including those of chemical, microbial, and parasitic origin (several of which are also NTDs), at a recent international consultation [34]. The result of the consultation was a draft strategic framework for the assessment of burden of foodborne diseases, which included: the outline of a roadmap for assembling existing information on the burden of disease, and a time frame outlining the individual strategic activities in relation to the roadmap. In addition, the participants agreed on the contents of a standard protocol for foodborne disease burden studies at country level, including infectious and chemical causes.

One of the major recommendations was the establishment of the FERG, which is charged with implementing the recommendations of the consultation and estimating the global burden of foodborne diseases. The FERG has the comparative advantage of being able to base its structure and procedures upon an assessment of the functioning and impact of previous WHO groups described in this paper.

**Figure 1:**
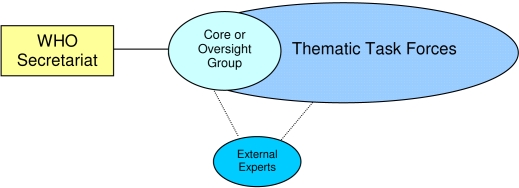
Composition and Structure of the Foodborne Disease Burden Epidemiology Reference Group (FERG)

It follows that:

- Similar to other initiatives where external reference groups have provided accurate and transparent estimates, the FERG's tasks include the assembly and appraisal of existing epidemiological information, the recommendation and/or commissioning of future work, the provision of burden of disease estimates, and the development of tools for countries to conduct burden of foodborne disease studies.
- A consultation to brainstorm the strategic way forward underpinned the planning of all aspects of FERG. The terms of reference and standard operating procedures for FERG were developed in partnership with the UN Food and Agricultural Organization, as well as external and WHO in-house scientists who were considered potential future members of FERG. This facilitates ownership of all mechanisms and clarity of roles of members.
- The members of FERG were nominated by the WHO Director-General following a widely distributed call for applications to governments, in the scientific press, and through other professional networks. This ensured a gender- and geographically balanced selection from a large pool of scientists.
- Due to the multi-factorial nature of foodborne diseases, the FERG is highly multidisciplinary and includes a large number of members. It therefore operates through a Core or Oversight Group as well as a number of different Task Forces (Figure 1)—a modus operandi that was very successful in the MERG.
- The high-level media coverage and advocacy-focused thinking of the NTD STAG is a model to be emulated. A detailed communication strategy has been developed that covers internal and external information sharing and mechanisms for accountability as well as all aspects of advocacy for FERG. Moreover, some key stakeholders (including consumer groups and donors) will be invited to provide input at the first formal meeting of FERG in November 2007, and a more comprehensive dedicated stakeholder meeting is planned for 2008.
- WHO is assembling an alliance of funding agencies and in-kind supporters for FERG, thus ensuring that no individual institution, foundation, or government may exert undue influence on this initiative. Although WHO has already made and will continue to make considerable financial investments in FERG, the Organization is currently discussing additional funding options with a number of governmental and non-governmental donors, as it will require approximately US$6 million over five years to complete the work.
- The FERG is expected to provide a Global Report and Atlas on the Burden of Foodborne Diseases as well as a series of journal papers. As in other initiatives, these products will be peer reviewed by scientists outside FERG to ensure highest quality and policy impact.

The Core or Oversight Group consists of scientists from each of the areas outlined in the task forces and is charged with monitoring and appraising the technical and epidemiological work of all task forces. The core group is chaired by a scientist with extensive international experience in both foodborne diseases and burden of disease methodology. Additional external experts can be called upon to join the FERG on an ad hoc basis to supplement the skills required.

FERG Task Forces are the executing arm of the FERG and conduct burden of disease work in the following areas:

- Task Force 1: Infectious diseases (sub-groups on enteric diseases and other infectious diseases, including parasites);
- Task Force 2: Chemicals and toxins;
- Task Force 3: Source attribution; and
- Task Force 4: Country Burden of Disease protocols

The Task Force on source attribution is unusual among any of the burden of disease efforts described in this paper. It is charged with identifying the proportion of disease burden that is directly due to food contamination, and will aim to isolate the specific food sources responsible. A specific Task Force on Country Burden of Disease protocols will develop user-friendly tools for countries to conduct their own burden of foodborne disease studies, thus enabling them to monitor progress of their food safety standards and interventions.

In addition to linking with other in-house and external partners, the FERG is also collaborating closely with major GBD initiatives, including the Institute for Health Metrics and Evaluation (IHME) in Seattle funded by the Bill and Melinda Gates Foundation. This Institute, which is led by Christopher Murray, has launched an initiative to provide an update of the GBD for the year 2005. In this initiative the Institute collaborates closely with WHO staff, while scientists leading burden of disease areas at IHME are also a nominated FERG members.

The FERG secretariat has held several preparation meetings with partners and will commence its Task Force work in November 2007. It is operating within a time frame of three to five years for the delivery of clearly identified milestones and products and is accountable to the WHO Director-General. WHO hopes that international interest in this initiative—which will ultimately help countries to estimate the magnitude of foodborne illness and assess progress with food safety interventions—will be high. Such interest was already signalled by Member States in the World Health Assembly in 2000, when countries acknowledged foodborne diseases as an important cause of illness and death worldwide and identified prevention and control of foodborne diseases as a public health priority [35].

## Synergy, Not Competition

The burden of disease initiatives described in this paper vary considerably in their structure and procedures, but they are linked by a common thread of strong internal and external collaborations. Given the different nature of the diseases described and the often multiple purposes of the groups established, it would not be prudent to prescribe a blueprint or “best practice” model for external WHO reference groups. For this reason, we have refrained from providing a detailed tabulated summary contrasting specific tactics and approaches that worked well and should be replicated with those that did not work well and should be avoided. The lessons learnt by these groups, however, are helpful in providing focus for the establishment and management of reference groups at WHO. It is apparent that WHO should aim for clarity of the purpose, roles, and procedures applied to the reference groups it convenes. This should include transparent selection procedures for experts, involvement of all stakeholders and partners in the process, and clear communication with constituents.

One emerging theme from these analyses is the identification of WHO (together with its UN partner organizations) as a natural coordinator of global efforts to assemble and describe health information due to its universally acknowledged convening mandate and power, its global platform and visibility, and its international credibility as a technical public health leader. This was emphasized by the late WHO Director-General Lee Jong-wook, who affirmed WHO's commitment following the high-impact CHERG publications by pledging to “play the leadership role…to monitor progress and hold the broader public health and development community accountable for reducing child and maternal mortality” [36]. The scientific community represented in the high-level panel on health statistics [16] upheld this notion by requesting WHO to continue to provide evidence-based health information for policy planning. An important addition to this was the recommendation that WHO should produce health information through work with external reference groups.

This is sound advice, and some of the examples described in this paper are testimony to the effectiveness of this approach, which combines independent expert advice and WHO's leadership capacity as indispensable elements when aiming for health information that is to be globally accepted and valued. WHO should therefore not be seen as the original provider of health information, but as a global navigator in the assembly, appraisal, and dissemination of such information. In doing so, it is paramount that WHO works synergistically with partners rather than in competition with other institutions. WHO must seize the increasing global interest in health information as well as recognizing the value and contribution of new initiatives, including IHME, to unite partners aiming for the same goal. This, however, does not absolve WHO from its unique responsibilities in the area of health information reporting, articulated by one of the fathers of the GBD, Christopher Murray, who emphasized WHO's “critical mandate to provide meaningful comparable information on outcomes to the world and to empower people with information that is central to their wellbeing” [37]. WHO's leading role in this regard could not be expressed more clearly.
